# Change of 0.34Cr-1Ni-Mo-Fe Steel Dislocation Structure in Plasma Electrolyte Hardening

**DOI:** 10.3390/ma14081928

**Published:** 2021-04-12

**Authors:** Bauyrzhan Rakhadilov, Zarina Satbayeva, Sherzod Ramankulov, Nurdaulet Shektibayev, Laila Zhurerova, Natalya Popova, Gulzhaz Uazyrkhanova, Zhuldyz Sagdoldina

**Affiliations:** 1 Research Center Surface Engineering and Tribology, Sarsen Amanzholov East-Kazakhstan University, Ust-Kamenogorsk 070000, Kazakhstan; rakhadilovb@mail.ru (B.R.); leila_uka@mail.ru (L.Z.); sagdoldina@mail.ru (Z.S.); 2 Department of Physics, Khoja Akhmet Yassawi International Kazakh-Turkish University, Turkestan 161200, Kazakhstan; sherzod.ramankulov@ayu.edu.kz (S.R.); nurdaulet_86@mail.ru (N.S.); 3 Physics Laboratory, Tomsk State University of Architecture and Building, 634003 Tomsk, Russia; natalya-popova-44@mail.ru; 4 Department of Physics, D. Serikbayev East Kazakhstan Technical University, Ust-Kamenogorsk 070000, Kazakhstan; guazyrhanova@mail.ru

**Keywords:** dislocation structure, steel, hardening, α-phase, martensite, carbide, cementite dislocation density

## Abstract

This work deals with the study of changes in the dislocation structure and quantitative characteristics, as well as morphological components, of 0.34Cr-1Ni-Mo-Fe steel before and after plasma electrolytic hardening. According to the electron microscopic studies of the fine structure of 0.34Cr-1Ni-Mo-Fe steel before and after plasma electrolytic hardening, 0.34Cr-1Ni-Mo-Fe steel is a multiphase material containing an α-phase, a γ-phase (retained austenite), and a cementite and carbide phase. It was revealed that, morphologically, the α-phase in the initial state, generally, is present in the form of: lamellar pearlite with a volume fraction of 35%, a ferritocarbide mixture with a volume fraction of 45%, and fragmented ferrite with a volume fraction of 20% of the material. After surface hardening, the morphological components of the structure changed: packet–lamellar martensite with volume fractions of 60% and 40%, 5% and 7% of γ-phase as residual austenite in the crystals of packet–lamellar martensite, 0.6% and 1.5% of cementite in crystals of packet–lamellar martensite, and 0.15% and 0.35% of complex carbide M_23_C_6_ in crystals of packet–lamellar martensite, respectively, were observed. The quantitative characteristics of the dislocation structure were estimated by the following calculated indices of packet and lamellar martensite: scalar (ρ) and excess (ρ±) density of dislocations, the value of the curvature-torsion of the crystal lattice (χ), the amplitude of long-range internal stresses (σd), and the amplitude of shear stresses (σ_L_), according to which the plastic nature of the bending-torsion of the crystal lattice was confirmed (σ_L_ > σd).

## 1. Introduction

The heating of metals in electrolytic plasma during the anodic or cathodic process, due to the favorable combination of the high temperature of the active electrode and the flow of electrical discharges in the vapor–gas envelope between the electrodes, allows us to carry out a number of processes of high-speed local thermal and chemical heat treatments on steel parts [1,2,3]. These processes include: plasma electrolytic oxidation (PEO) [4], plasma electrolytic hardening (PEH) [5], and plasma electrolytic saturation (PES) processes such as plasma electrolytic nitriding (PEN) [6], plasma electrolytic carburizing (PEC) [7], etc. Among them, PEH is of great interest. PEH involves rapid (short-term) heating of the surface to high temperatures with subsequent cooling [5,8,9]. This method favorably differs, in the simplicity of its technological process and its efficiency, from other types of surface heat treatment, such as hardening by high frequency current, laser or electron beams, plasma jets, etc. Nevertheless, the known PEH methods have certain disadvantages associated with a low heating rate. The PEH methods described in [10,11,12] have a heating rate of ≈200 °C/s. This does not allow for the effect of plasma hardening to be achieved, which is widely used in production [13]. Thus, the heating rate has a significant effect on the size of the recrystallized grain, causing it to grind. Short-term exposure of steel to the hardening temperature range and the occurrence of phase transformations at temperatures exceeding equilibrium lead to the material obtaining mechanical properties that differ from those of steel quenched using traditional heat sources. The use of rapid heating, which helps yield a finer structure of the hardened steel, makes it possible to obtain a more favorable combination of strength and toughness properties. This is especially important for increasing the service life of gears. Therefore, at present, surface thermal hardening is one of the most effective ways to increase the service life of loaded elements of machines and mechanisms made of alloyed medium-carbon steels.

According to the preliminary results of [14,15,16], it is possible to increase the heating rate of the cathode to 400–500 °C/s by changing the size and shape of the anode and the composition of the electrolyte. This makes it possible to obtain a modified layer 0.7–2 mm thick that contributes to an increase in the hardness of alloy steels by 2–2.5 times and wear resistance by 2–2.2 times. The structure of alloyed steels consists of fine martensite formed during surface hardening by a plasma jet or laser or electron beams [17]. Structural transformations generally correspond to those occurring during bulk quenching, however, high heating and cooling rates cause a change in the ratios between structural components, a change in their morphology due to dispersion, the formation of new phases, an increased defectiveness of the crystal structure, and a change in the dislocation structure of steels [18]. The influence of surface hardening by a plasma jet or laser or electron beams on the structural-phase transformations of steels has been studied in depth. Nevertheless, the effect of PEH on structural-phase transformations in steels has been studied poorly. In this case, the effect of high-speed PEH (400–500 °C/s) on the dislocation structure of steels is absent from the literature.

The aim of this work is to study the morphology of phase transformations and quantitative characteristics of the dislocation structure of the modified surface layers of alloyed 0.34Cr-1Ni-Mo-Fe steel during PEH.

## 2. Materials and Methods

The object of the study was 0.34Cr-1Ni-Mo-Fe steel (GOST 8479-70) with a chemical composition of: C 0.3–0.4%, Si 0.17–0.37%, Mn 0.5–0.8%, Ni 1.3–1.7%, S up to 0.035%, P up to 0.03%, Cr 1.3–1.7%, Mo 0.2–0.3%. This material was chosen because this steel is widely used for the manufacture of heavily loaded gear wheels.

Electrolytic plasma hardening of steel samples was carried out in the cathodic mode in an electrolyte-containing aqueous solution of 20% sodium carbonate and 10% carbamide. To heat the sample to the quenching temperature, a voltage of 320 V was applied between the electrodes for 2 s, then the voltage was turned off and the sample was rapidly cooled due to heat removal from the surface to the base of the material and due to the flowing electrolyte. Detailed descriptions of the installation and technological processing are given in [14,19,20].

The morphological analysis of the samples treated in electrolyte plasma was carried out on a JSM-6390LV scanning electron microscope (JEOL, Tokyo, Japan), with an INCAEnergy energy dispersive microanalysis attachment from OXFORD Instruments (Abingdon, UK). The microhardness of steel samples was measured on a PMT-3M instrument in accordance with GOST 9450-76 (LOMO, St. Petersburg, Russia), with loads on the indenter of P = 1 N and a holding time of 10 s. Abrasive wear tests on the samples were carried out using a “flat surface-rotating disc” scheme according to ASTM G65. Wear resistance was assessed by a weight loss method. Weight loss was measured by the gravimetric method using an ADV-200 analytical (TechnoAnalit, Ust-Kamenogorsk, Kazakhstan) balance with an accuracy of 0.0001 g.

The morphology of the fine structure was studied by transmission electron microscopy (TEM) on thin foils using an EM-125 electron microscope (SEDP, Sumy, Ukraine) at an accelerating voltage of 125 kV and magnification from 8000 to 50,000 times. The determination of the size and volume fraction of the carbide phases, as well as α- and γ-phases, was carried out using images confirmed by microdiffraction patterns and dark-field images obtained in the reflections of the corresponding phases. The phases were identified according to standard methods [21]. For this, microdiffraction patterns were used, calculated from the tabular values of the crystal lattice parameters. The results obtained by electron microscopy were compared with the literature and processed by statistical methods.

The quantitative parameters were estimated by the planimetric method of determining the volume fraction from random sections based on measuring the fraction of the foil area Ps occupied by a certain type of dislocation substructure (DSS). According to this method, the areas of images of each DSS type on the observation plane were measured. Then the values of such areas were summed up. The resulting sum was divided by the studied area of the observation plane. The scalar dislocation density was measured by the secant method with a correction for the invisibility of dislocations [22,23]. A rectangular mesh was used as a test line.

(1) $$\rho =\sum_{i=1}^{Z}P_{\mathrm{Vi}}\rho_{i}$$

where ρ_i_ is the scalar density of dislocations in a certain DSS type; P_Vi_ is the volume fraction of the material occupied by this type of DSS; Z is the number of DSS types.

The excess dislocation density $\rho_{\pm }{=\rho }_{+}{+\rho }_{-}^{}$ ($\rho_{+}$ and $\rho_{-}^{}$ are the densities of positively and negatively charged dislocations, respectively) was measured locally along the misorientation gradient [24,25,26]:

(2) $$\rho_{\pm }=\frac{1}{b}\frac{\partial \phi }{\partial l}$$

where b is the Burgers vector of dislocations; $\frac{\partial \phi }{\partial l}$ is the gradient of the curvature of the foil or the curvature-torsion of the crystal lattice (χ). The value $\chi =\frac{\partial \phi }{\partial l}$ was determined by shifting the extinction contour (∆l) at a controlled angle of inclination of the foil (∆φ) in the microscope column using a goniometer.

The magnitude of the internal stress fields, following the calculation scheme [27], can be estimated as follows:

(3) σ=G⋅t⋅χ

where G is the shear modulus of the material; t is the thickness of the foil (for EM-125 electron microscope, t ~ 200 nm); χ is the curvature-torsion of the crystal lattice.

## 3. Results and Discussion

**Figure 1:**
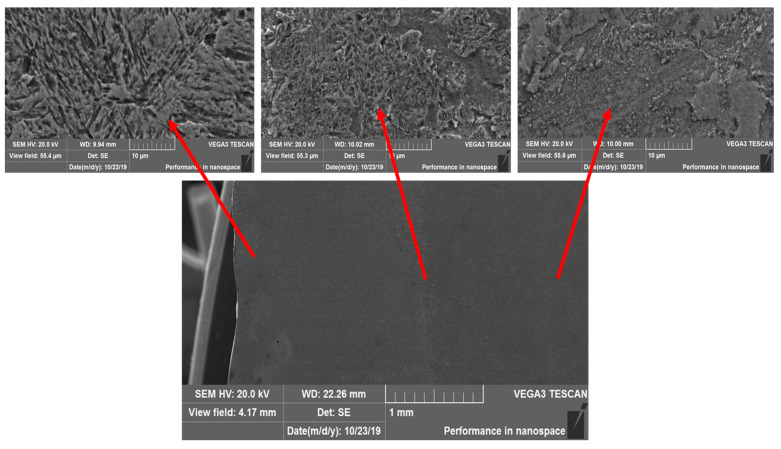
Microstructure of 0.34Cr-1Ni-Mo-Fe steel.

Figure 1 shows fragments of the microstructure, obtained using a scanning electron microscope, of a transverse section of 0.34Cr-1Ni-Mo-Fe steel after PEH, where the zoning of the structure characteristic of surface hardening is observed. PEH of steel resulted in modification of the surface layer of the sample. The layer structure changes with distance from the sample surface. The image of a transverse microsection shown in Figure 1 was obtained using a scanning electron microscope at a relatively low magnification. The modified surface layer consisting of martensite is well distinguished on the transverse microsection, the thickness of which is ~1.7–2 mm on average. Furthermore, deeper into the sample, there is a transitional layer, and the layer (zone) of the main material.

**Figure 2:**
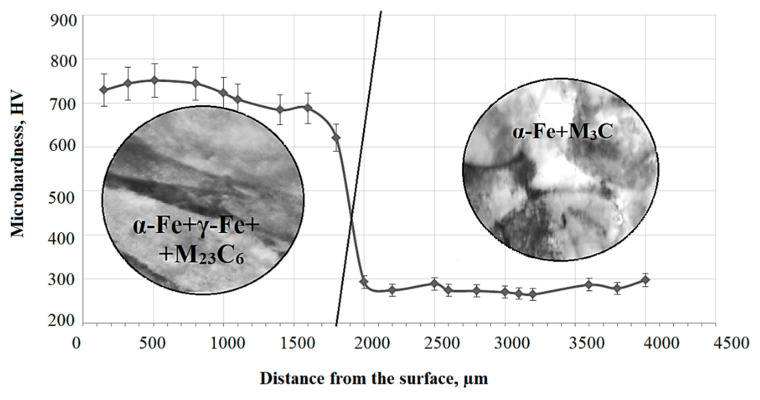
Distribution of microhardness along the depth of 0.34Cr-1Ni-Mo-Fe steel after plasma electrolytic hardening (PEH).

One of the most important properties of the surface layer, which greatly affects wear resistance, is hardness. Figure 2 shows the change in microhardness over depth of a sample treated with PEH. Microhardness data confirmed the formation of a martensitic structure. Microhardness increases significantly near the surface. The nature of the transition zone includes a smooth transition from the hardened layer to the base, while the microhardness of the transition zone is slightly less than that of the base, and the microhardness of the base does not change.

Table 1 shows data on the structure and tribological characteristics of steel after PEH. The experimental data clearly illustrate the correlation between the structural and tribological characteristics of quenched samples. From the data in Table 1, a significant increase in the tribological properties of the steel after PEH can be seen.

### 3.1. The Structure of the Initial State of 0.34Cr-1Ni-Mo-Fe Steel

Regardless of the processing of the sample and the place of study, the main phase component (matrix) of 0.34Cr-1Ni-Mo-Fe steel is the α-phase of various degrees of alloying. According to the literature data [28], parameters of the phases’ crystal lattices are given in Table 2.

The α-phase has a body-centered cubic (BCC) lattice (Table 2) and is a solid solution based on iron atoms of interstitial (primarily C and others) and substitution (Cr, Mn, Mo, etc.) elements simultaneously. Regardless of the processing of the material and the place of examination of the sample, it always constitutes the bulk of the material. Morphologically, the α-phase is generally present in the form of: (1) lamellar pearlite with a volume fraction of 35%, (2) a ferrite–carbide mixture, and (3) fragmented ferrite with a volume fraction of 45% and 20% in the material, respectively.

**Figure 3:**
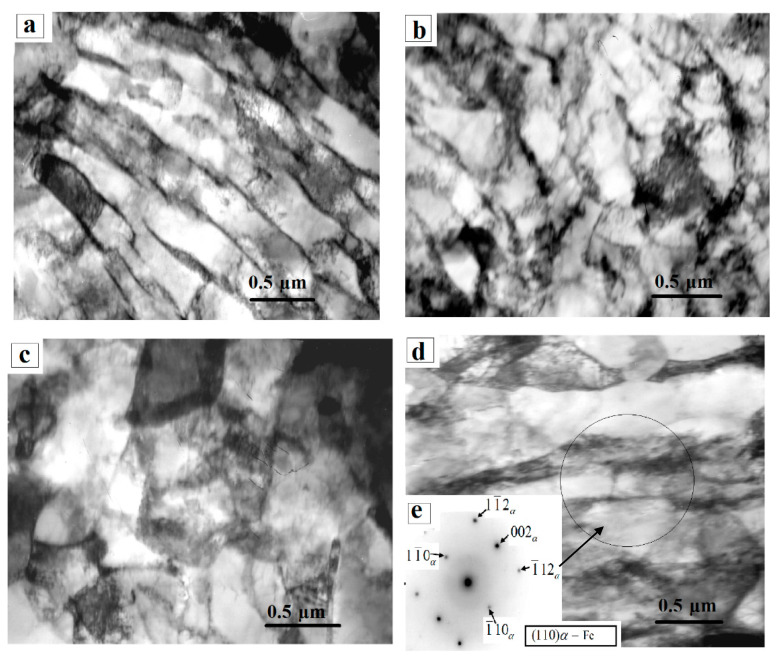
Electron microscopic image of the dislocation structure of 0.34Cr-1Ni-Mo-Fe steel in the initial state. (**a**) lamellar pearlite, (**b**) primary fragmented perlite, (**c**) ferrite-carbide mixture, (**d**) fragmented ferrite, (**e**) microdiffraction pattern.

An electron microscope image of the morphological components of the phases in 0.34Cr-1Ni-Mo-Fe steel in the initial state and the quantitative characteristics of the dislocation structure in them are shown in Figure 3 and Table 3, respectively. The morphological components of the surface of 0.34Cr-1Ni-Mo-Fe steel in the initial state are lamellar pearlite, ferrite–carbide mixture, and fragmented ferrite. Lamellar pearlite presents in the form of almost ideal (Figure 3a) pearlite formed by alternating parallel plates of ferrite (α-phase) and cementite and fragmented pearlite (or of primary fragmented perlite) (Figure 3b) with the preserved structure of pearlite colonies. The average value of scalar dislocation density inside the fragments is 1.5 × 10^10^ cm^−2^. The volume fraction of pearlite in the initial state of 0.34Cr-1Ni-Mo-Fe steel is 35%, the volume fraction of ideal pearlite is 10%, and that of fragmented pearlite is 25%.

The ferrite­–carbide mixture is the second morphological component of the structure of 0.34Cr-1Ni-Mo-Fe steel in the initial state (Figure 3c). Cementite is located not only along the boundaries of fragments, but also at their joints, forming a so-called cementite network. Accordingly, such areas of the material structure are actually a mixture of grains of the α-phase and cementite, and therefore it is called a “ferritic carbide mixture”. Such a dislocation structure is formed during the secondary fragmentation of pearlite. The volume fraction of this structure in the material is ~45%. The average value of the scalar dislocation density within fragments of the α-phase is 2.25 × 10^10^ cm^−2^.

The third morphological component of 0.34Cr-1Ni-Mo-Fe steel’s structure in the initial state is fragmented ferrite (Figure 3d). According to the microdiffraction pattern (obtained from the area marked with a circle, from the plane (110) of the α-phase) in Figure 3, the structure of the fragmented ferrite is the α-phase, in which the direction of the fragments is preserved like pearlite colonies, which is typical of the structure of the next stage of pearlite transformation. Apart from the reflections of the α-phase, reflections of cementite and other carbide phases were not found. The volume fraction of this structure in the material is 20%. The average value of the scalar dislocation density inside fragments of the α-phase is 2.95 × 10^10^ cm^−2^ [29,30,31].

### 3.2. Structure of 0.34Cr-1Ni-Mo-Fe Steel after PEH

Plasma electrolytic hardening of the 0.34Cr-1Ni-Mo-Fe steel’s surface led to significant qualitative and quantitative changes in the steel structure, namely, to a change in the phase composition and the list of phases present, as well as their morphology. Thus, both the phase composition and the fine structure of the steel in the near-surface layer of the sample differ significantly from the initial state.

**Figure 4:**
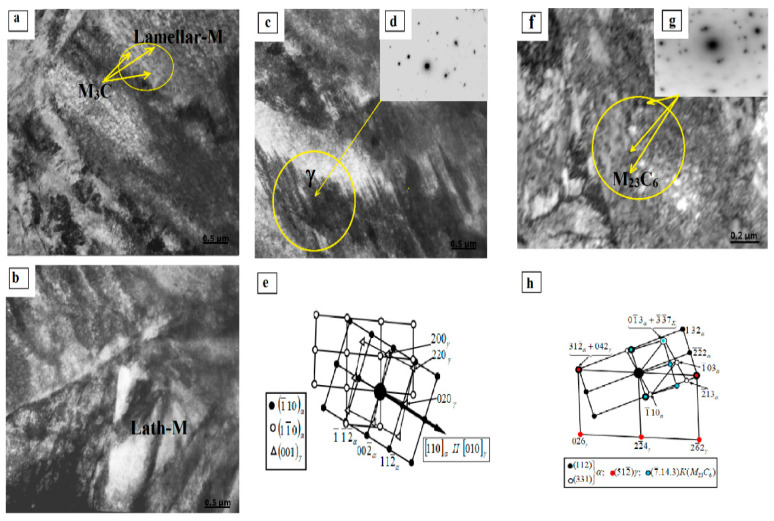
Electron microscopic image of the dislocation structure of the surface of 0.34Cr-1Ni-Mo-Fe steel sample after surface hardening. (**a**) Lamellar martensite, (**b**) Lath (packet) martensite, (**c**–**e**) γ–Fe residual austenite, its microdiffraction pattern and indexed schema, (**f**–**h**) M_23_C_6_ are carbides in the form of a colony, its microdiffraction pattern and indexed schema.

Surface hardening led to the formation of a martensitic structure. The morphology of the fine structure is represented by a mixture of packet (or lath) and lamellar martensite, i.e., packet–lamellar martensite, γ-phase as residual austenite, and particles of the carbide phase, namely, cementite and complex carbide M_23_C_6_. A typical electron microscopic image of the fine structure of the surface of the 0.34Cr-1Ni-Mo-Fe steel sample after surface hardening is shown in Figure 4, where Lath-M is packet (lath) martensite (Figure 4b); Lamellar-M is lamellar martensite (Figure 4a); γ is Fe-residual austenite and its microdiffraction pattern (Figure 4d), obtained from the area marked with a circle in Figure 4, as well as its indicated diagram (the arrow in Figure 4e marks the coinciding directions [110]α and [010]γ, while the condition ($\bar{1}10$)α II (001)γ is the Kurdyumov–Sachs ratio); M_3_C is cementite (Figure 4a), the area of formation of cementite needles is marked with a circle; M_23_C_6_ are carbides in the form of a colony (Figure 4f–h).

The morphological components of the α-phase of 0.34Cr-1Ni-Mo-Fe steel after PEH and the quantitative characteristics of the dislocation structure in them according to calculations and data of electron microscopy of the sample surface are given in Table 4.

According to statistically processed data obtained with an electron microscope, the following quantitative characteristics of the morphological components of the structure were determined: volume fractions, sizes, volume fractions of the distribution density of secondary phases and their localization, parameters of the fine structure of the material (scalar (ρ) and excess (ρ±) dislocation density), amplitudes of curvature-torsion of the crystal lattice (χ), and internal stresses (σ).

It is known that the bending-torsion of the crystal lattice can be plastic, in which case χ = χ_pl_, or elastic, in which case χ = χ_el_. Elastic–plastic bending-torsion is also possible (χ = χ_pl_ + χ_el_) when both sources of fields are present in the material. The type of bending-torsion of the crystal lattice is determined by calculating the excess dislocation density (ρ±) by the formula ρ± = χ/b, where b is the Burgers vector of α-iron. According to these calculations, it was found that in the packet martensite, the average value of the excess dislocation density (ρ±) is 2.06 × 10^10^ cm^−2^, while that of the lamellar martensite is 1.84 × 10^10^ cm^−2^. The excess dislocation density for the whole material is 1.97 × 10^10^ cm^−2^. Comparing the obtained values of ρ_±_ with the value of ρ in each structural component, it can be argued that in both the packet and lamellar martensite, the condition ρ > ρ± is fulfilled. This means that the amplitude of the curvature-torsion of the crystal lattice in the entire material is χ = χ_pl_ and that the bending-torsion of the crystal lattice is of a plastic nature, which means that PEH does not lead to destruction of the material.

The bending-torsion of the crystal lattice led to the formation of long-range stresses. These are internal stresses that have arisen in places within the material with an excessive density of dislocations. These are local (or momentary) internal stresses (σ_d_). Under the condition χ = χ_pl_, the amplitude of local internal stresses (σ_d_) was calculated: in packet martensite it is equal to 285 MPa, in plate martensite it is 270 MPa, and in general for the material it is equal to 280 MPa. Further internal stresses were also identified, which are formed by the dislocation structure and are called shear stresses (σ_L_). The calculations show that σ_L_ = 390 MPa in packet martensite, 345 MPa in plate martensite, and 370 MPa in the material as a whole.

The calculated quantitative parameters of the fine structure of 0.34Cr-1Ni-Mo-Fe steel before and after surface hardening for each morphological component (packet and lamellar martensite), as well as for the material as a whole, are presented in Table 5.

As can be seen from Table 5, firstly, all quantitative parameters of packet martensite are higher than those of lamellar. Secondly, in the entire material after surface hardening, as in the initial state, the following conditions are met: ρ > ρ± and σ_L_ > σ_d_. This means that bending-torsion (distortion) of the crystal lattice of 0.34Cr-1Ni-Mo-Fe steel after surface hardening is also purely plastic in nature, which will not lead to the formation of microcracks in the material.

## 4. Conclusions

By the evaluation of electron microscopic studies and measurement analysis, it was found that:

(1) Surface hardening led to the formation of packet–lamellar martensite with volume fractions of 60% and 40%, respectively, and γ-phase in the form of residual austenite with fractions of 5% and 7% in packet–lamellar martensite crystals, respectively, as well as particles of the carbide phase, namely, cementite with fractions in crystals of lamellar martensite of 0.6% and 1.5%, respectively, and complex carbide M_23_C_6_ with fractions in crystals of packet–lamellar martensite of 0.15% and 0.35%, respectively;
(2) The scalar dislocation density (ρ) of packet and lamellar martensite after PEH are equal to 3.78 × 10^10^ cm^−2^ and 3.0 × 10^10^ cm^−2^, respectively, which is 1.5 times higher than in the initial state; the amplitude of the long-range internal stresses of the packet and lamellar martensite are σ_d_ = 285 MPa and σ_d_ = 270 MPa, and the amplitude of the shear stresses of the packet and lamellar martensite have the values σ_L_ = 390 MPa and σ_L_ = 345 MPa, that is, the condition σ_L_ > σ_d_ is met, which in turn confirms the plastic nature of the bending-torsion of the crystal lattice.

## Figures and Tables

**Table 1:** Experimental data on the structure and tribological characteristics of 0.34Cr-1Ni-Mo-Fe steel.

Material State	Characteristics						
	Phase State	Hµ, HV	f	j, 10^−4^ mm^3^/Nm	V, µm^3^	∆m, mg	Ra, μm
The Initial State	α-Fe, M_3_C	283	0.44	1.08	1.05	36.03	0.199
After PEH	α-Fe, γ-Fe, M_23_C_6_	742	0.37	0.32	0.6	20.63	0.446

Note: Hµ—microhardness; f—coefficient of friction; j—wear rate; V—wear volume; ∆m—weight loss due to abrasive wear; Ra—roughness.

**Table 2:** Type, space group, and parameters of crystal lattices of α-phase, γ-phase, and carbides.

Phase	Crystal Lattice Type	Space Group	Crystal Lattice Parameters, nm		
			a	b	c
α-Fe	BCC	*Im3m*	0.28664		
γ-Fe	FCC	*Fm3m*	0.3573		
M_3_C	Orthorhombic	*Pnma*	0.5080	0.6774	0.4520
M_23_(C)_6_	FCC	*Fm3m*	1.0585		

Note: M—Fe, Cr, Ni, Mn.

**Table 3:** Morphological components of 0.34Cr-1Ni-Mo-Fe steel in the initial state and quantitative characteristics of the dislocation structure in them.

Morphological Component of the Steel Matrix	Volume Fraction in the Steel Matrix, P_V_ ± 5, %	Average Scalar Dislocation Density, ρ × 10^10^ ± 0.01 × 10^10^, cm^−2^
Sample surface		
Lamellar perlite	35.00	1.50
Ferrite–carbide mixture	45.00	2.25
Fragmented ferrite	20.00	2.95
In the material	100.00	2.20

**Table 4:** Morphological components of the α-phase of 0.34Cr-1Ni-Mo-Fe steel after PEH and quantitative characteristics of the dislocation structure in them.

Quantitative Characteristics of the Dislocation Structure	Packet Martensite	Lamellar Martensite
Volume fraction in the steel matrix, P_V_, %	60.00	40.00
Average size of packs or plates of martensite, d, µm	0.25	1.10
Volume fraction of cementite, δ,%	0.60	1.50
Average particle size of cementite, d, nm	12 × 32	20 × 80
Fraction of retained austenite in crystals of the α-phase, δ,%	5.00	7.00
Volume fraction of M_23_C_6_-type carbides, δ,%	0.15	0.35
Average particle size of M_23_C_6_-type carbides, d, nm	8.00	16.00

**Table 5:** Quantitative characteristics of the dislocation structure of the morphological components of the α-phase of 0.34Cr-1Ni-Mo-Fe steel after PEH.

Phase	Average Quantitative Parameters of the Dislocation Structure					
	P_V_ ± 5%	ρ± 0.1 × 10^10^, cm^−2^	ρ±, cm^−2^	χ, cm^−1^	σ_L_, MPa	σ_d_, MPa
After Surface Hardening						
Packet martensite	60%	3.78 × 10^10^	2.06 × 10^10^	515	390	285
Lamellar martensite	40%	3.00 × 10^10^	1.84 × 10^10^	460	345	270
In the material	100%	3.47 × 10^10^	1.97 × 10^10^	495	370	280
Initial State						
In the material	100%	2.20 × 10^10^	~0	~0	295	~0

## Data Availability

Data are contained within the article.

## References

[1] Duraji V.N. (2010). Chemical-thermal treatment of metals with heating in electrolytic plasma. Actual Conf. Surf. Treat. Technol. M.

[2] Tyurin Y.N., Pogrebnyak A.D. (2001). Electric heating using a liquid electrode. Surf. Coat. Technol..

[3] Yerokhin A.L., Nie X., Leyland A., Matthews A., Dowey S.J. (1999). Plasma electrolysis for surface engineering. Surf. Coat. Technol..

[4] Agureev L., Savushkina S., Ashmarin A., Borisov A., Apelfeld A., Anikin K., Tkachenko N., Gerasimov M., Shcherbakov A., Ignatenko V. (2018). Study of plasma electrolytic oxidation coatings on aluminum composites. Metals.

[5] Belkin P.N., Kusmanov S.A. (2016). Plasma electrolytic hardening of steels: Review. Surf. Eng. Appl. Electrochem..

[6] Kusmanov S.A., Smirnov A.A., Silkin S.A., Belkin P.N. (2016). Increasing wear and corrosion resistance of low-alloy steel by anode plasma electrolytic nitriding. Surf. Coat. Technol..

[7] Tarakci M., Korkmaz K., Gencer Y., Usta M. (2005). Plasma electrolytic surface carburizing and hardening of pure iron. Surf. Coat. Technol..

[8] Shadrin S.Y., Belkin P.N. (2012). Analysis of models for calculation of temperature of anode plasma electrolytic heating. Int. J. Heat Mass Transf..

[9] Dayança A., Karacaa B., Kumruoğlub L.C. (2017). The cathodic electrolytic plasma hardening of steel and cast iron based automotive camshafts. Acta Phys. Pol. A.

[10] Belkin P.N., Kusmanov S.A., Smirnov A.A. (2016). Plasma electrolytic hardening and nitrohardening of medium carbon steels. Mater. Sci. Forum.

[11] Luk S.F., Leung T.P., Miu W.S., Pashby I. (1999). A study of the effect of average preset voltage on hardness during electrolytic surface-hardening in aqueous solution. J. Mat. Proc. Technol.

[12] Bejar M.A., Henriquez R. (2009). Surface hardening of steel by plasma-electrolysis boronizing. Mater. Des..

[13] Popova N.A., Nikonenko E.L., Nikonenko A.V., Gromov V.E. (2019). Effect of electrolytic-plasma nitrocarburizing on the structural and phase state of ferrite-pearlitic steels. Steel Transl..

[14] Rakhadilov B.K., Satbayeva Z.A., Bayatanova L.B., Kilyshkanov M.K., Kalibayev K.A., Kochneva A.K. (2019). Influence of electrolyte-plasma surface hardening on the structure and properties of steel 40KhN. J. Phys. Conf. Ser..

[15] Rakhadilov B., Satbayeva Z., Baizhan D. (2019). Effect of electrolytic-plasma surface strengthening on the structure and properties of steel 40KhN. METAL.

[16] Satbayeva Z.A., Baizhan D.R., Kenesbekov A.B. Features of structure formation in 40KhN steel during electrolyte-plasma surface hardening. Proceedings of the 11 International Symposium “Powder Metallurgy: Surface Engineering, New Powder Composite Materials, Welding”.

[17] Safonov E.N. (2014). Plasma Hardening of Machine Parts: Monograph.

[18] Korotkov V.A. (2012). Surface Plasma Hardening.

[19] Rakhadilov B.K., Skakov M.K., Sagdoldina Z.B., Kenesbekov A.B. (2018). Method of Surface Hardening of Steel Products: Patent for Invention of the Republic of Kazakhstan.

[20] Rakhadilov B.K., Zhurerova L., Pavlov A. (2016). Method of electrolyte-plasma surface hardening of 65G and 20GL low-alloy steels samples. IOP Conf. Ser. Mater. Sci. Eng..

[21] Saltykov S.A. (1976). Stereometric Metallography. M. Metallurgyl..

[22] Hirsch P., Howie A., Nicholson P., Pashley D., Whelan M. (1968). Electron microscopy of thin crystals. Mosc. Mir.

[23] Utevsky L.M. (1973). Diffraction electron microscopy in metal science. Mosc. Metall..

[24] Koneva N.A., Lychagin D.V., Teplyakova L.A. (1984). Crystal lattice reversals and stages of plastic deformation//Experimental research and theoretical description of disclinations. Leningr. FTI.

[25] Koneva N.A., Lychagin D.V., Zhukovsky S.P., Kozlov E.V. (1985). Evolution of dislocation structure and stages of plastic flow of polycrystalline iron-nickel alloy. FMM.

[26] Koneva N.A., Kozlov E.V. (1982). The nature of substructural hardening. Izv. Vuzov. Phys..

[27] Gromov V.Y., Kozlov E.V., Bazaykin V.I., Zellermaer V.Y., Ivanov Y.F., Ignatenko L.N., Zakirov D.M. (1997). Physics and mechanics of drawing and volumetric stamping. Mosc. Nedra.

[28] Gulyaev A.P. (1977). Metallurgy. M. Metall..

[29] Popova N.A., Nikonenko Y.L., Nikonenko A.V., Gromov V.Y., Peregudov O.A. (2019). Influence of electrolyte plasma nitrocementation on the structural-phase state of ferrite-pearlite steels. Proceedings of higher educational institutions. Chernaya Metall..

[30] Popova N., Erygina L., Nikonenko E., Kalashnikov M., Skakov M. (2017). Structure and phase transformations in 0.34C-1Cr-1Ni-1Mo-Fe steel after electrolytic-plasma treatment. AIP Conf. Proc..

[31] Popova N., Erygina L., Nikonenko E., Skakov M. (2017). Phase composition of perlite steel modified by electrolyte plasma nitriding. AIP Conf. Proc..

